# Strategies to Treat Chronic Pain and Strengthen Impaired Descending Noradrenergic Inhibitory System

**DOI:** 10.3390/ijms20040822

**Published:** 2019-02-14

**Authors:** Ken-ichiro Hayashida, Hideaki Obata

**Affiliations:** 1 Doctorial Course in Medicine, Organ Function-Oriented Medicine, Akita University Graduate School of Medicine;1-1-1, Hondo, Akita-City, Akita 010-8543, Japan; hayashida@med.akita-u.ac.jp; 2 Center for Pain Management and Department of Anesthesiology, Fukushima Medical University; 1 Hikarigaoka, Fukushima-City, Fukushima 960-1295, Japan

**Keywords:** locus coeruleus, noradrenaline, descending inhibition, spinal cord, α_2_-adrenergic receptors, neuropathic pain, hypersensitivity, rats

## Abstract

Gabapentinoids (gabapentin and pregabalin) and antidepressants (tricyclic antidepressants and serotonin noradrenaline reuptake inhibitors) are often used to treat chronic pain. The descending noradrenergic inhibitory system from the locus coeruleus (LC) to the dorsal horn of the spinal cord plays an important role in the analgesic mechanisms of these drugs. Gabapentinoids activate the LC by inhibiting the release of γ-aminobutyric acid (GABA) and inducing the release of glutamate, thereby increasing noradrenaline levels in the spinal cord. Antidepressants increase noradrenaline levels in the spinal cord by inhibiting reuptake, and accumulating noradrenaline inhibits chronic pain through α_2_-adrenergic receptors in the spinal cord. Recent animal studies, however, revealed that the function of the descending noradrenergic inhibitory system is impaired in chronic pain states. Other recent studies found that histone deacetylase inhibitors and antidepressants restore the impaired noradrenergic descending inhibitory system acting on noradrenergic neurons in the LC.

## 1. Introduction

Although gabapentinoids (gabapentin and pregabalin, also known as voltage-dependent calcium channel α2δ subunit ligands) and antidepressants, such as tricyclic antidepressants (TCA) and serotonin noradrenaline reuptake inhibitors (SNRI), were not originally designed as analgesics, they have analgesic effects for chronic pain. These drugs have no substantial antinociceptive effects for acute pain but are considered first-line drugs of choice for treating neuropathic pain [1,2,3,4] and fibromyalgia [5]. Gabapentinoids and antidepressants use a common neuronal pathway to inhibit chronic pain, which includes the descending noradrenergic system from the locus coeruleus (LC) to the dorsal horn of the spinal cord. Gabapentinoids activate the LC whereas antidepressants inhibit the reuptake of noradrenaline in the synaptic cleft, both resulting in increased noradrenaline levels in the spinal cord. In this review, we discuss drug strategies to reinforce the descending noradrenergic inhibitory system in a chronic pain state based on experimental findings from animal models of neuropathic pain.

## 2. Descending Noradrenergic Inhibition from the LC

### 2.1. Physiological Role of the LC

In the central nervous system, all noradrenergic nuclei are located in the brainstem and are classified from A1 to A7. The largest noradrenergic nucleus, A6, also known as the LC, named over 200 years ago after the Latin word meaning “blue spot”, is located in the dorsal pons and contains more than 50% of all noradrenergic neurons [6,7]. LC neurons project to almost the entire central nervous system and are spatially subdivided by their efferent targets to regulate sensory gating and responses, including cognitive function (attention and memory), sleep and arousal, anxiety, and pain [8]. Although the ascending noradrenergic pathways from the dorsal LC can facilitate nociception, a large number of basic research studies suggest that the descending noradrenergic pathway from the ventral LC reduces spinal pain transmission [9,10]. In particular, large multipolar neurons in the ventral LC projecting to the dorsal horn of the spinal cord play an important role in endogenous analgesia [8,11].

### 2.2. Normal State

In the normal physiologic state, noradrenaline released from descending noradrenergic axons produces antinociceptive effects in the spinal dorsal horn via stimulation of the α_2_-adrenergic receptors, which are coupled with inhibitory G protein (Gi/o). Activation of presynaptic α_2_-adrenergic receptors on the primary afferents inhibits voltage-gated Ca^2+^ channels to reduce the release of excitatory neurotransmitters in the spinal cord. Activation of postsynaptic α_2_-adrenergic receptors on secondary sensory neurons in the spinal cord results in an opening of inwardly rectifying K^+^ channels to hyperpolarize cells, thereby reducing neuronal excitability [12]. Through these mechanisms, activation of the descending noradrenergic inhibitory pathway reduces spinal pain transmission.

### 2.3. Early Stage of Neuropathic Pain

**Figure 1:**
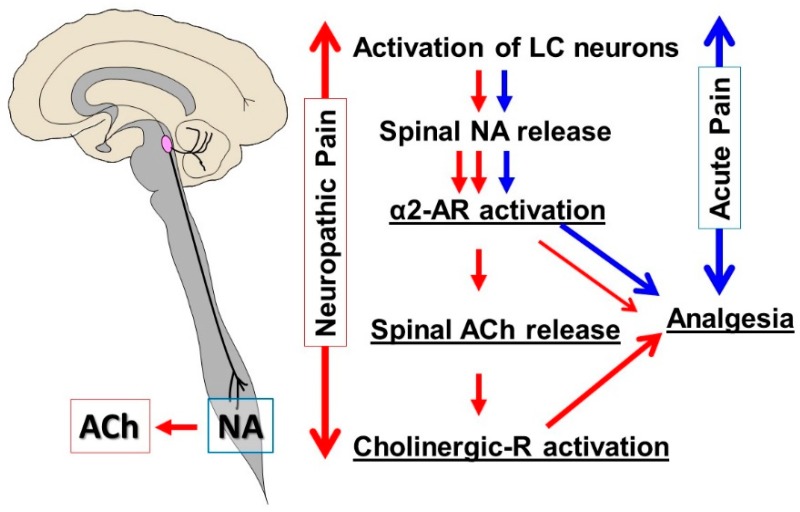
Locus coeruleus (LC) and descending noradrenergic inhibition. In a normal physiologic state (blue pathway), activation of LC neurons results in spinal noradrenaline (NA) release, which stimulates α2-adrenergic receptors (α2-AR) in the spinal cord to produce analgesia. In early-stage neuropathic pain following peripheral nerve injury (red pathway), noradrenergic axons sprout in the spinal cord, and the function of the α2-AR in the spinal cholinergic neurons changes from inhibition (Gi/o-coupling) to facilitation (Gs-coupling). Therefore, activation of LC neurons results not only in an increased release of NA but also the excitation of cholinergic interneurons to induce the release of acetylcholine (ACh) in the spinal cord, which is critical to the antihypersensitivity effect of spinal noradrenaline after nerve injury.

In rodents, at a relatively early stage of neuropathic pain following peripheral nerve injury (>2–3 weeks after injury), descending noradrenergic inhibition becomes profoundly effective against mechanical and thermal hypersensitivity [13,14]. This is due to the increased brain-derived neurotrophic factor (BDNF) in the spinal dorsal horn which, after nerve injury, fundamentally alters the structure and function of the descending noradrenergic pathway via the activation of tropomyosin receptor kinase B (trkB) [15,16]. On the activation of this pathway, noradrenergic fibers in the spinal dorsal horn sprout at dermatomes, surrounding the site of primary sensory input, allow for a more anatomically extensive release of noradrenaline. Furthermore, the function of the α_2_-adrenergic receptor in the spinal cholinergic neurons changes from inhibition (Gi/o-coupling) to facilitation (Gs-coupling); thus, spinally released noradrenaline excites cholinergic interneurons to induce acetylcholine release, which is critical to the antihypersensitivity effect of spinal noradrenaline after nerve injury (Figure 1). In addition, many drugs, including gabapentinoids, noradrenaline reuptake inhibitors, and clonidine, have been approved to treat neuropathic pain, activate, augment, or mimic the descending noradrenergic pathway to produce analgesia [17,18,19,20]. This suggests that the descending noradrenergic pathway is not only essential to endogenous analgesia but is also an important target for many drugs that have been approved to treat neuropathic pain.

### 2.4. Chronic Neuropathic Pain

When neuropathic pain turns into chronic pain, noradrenergic neurons in the LC become less responsive to noxious stimuli, leading to impaired endogenous analgesia. Astroglial glutamate dysregulation is critical to this impairment [21]. Among the various neurochemical inputs to the LC, glutamate is considered a primary excitatory regulator of noradrenergic neurons, acting through α-amino-3-hydroxy-5-methyl-4-isoxazolepropionic acid (AMPA) receptors [7]. Glutamate also inhibits its own release from the terminal via group 2 and 3 metabotropic glutamate receptors (mGluRs) [12]. In the central nervous system, two types of astroglial glutamate transporters, glutamate transporter-1 (GLT-1) and glutamate-aspartate transporter, regulate extracellular glutamate [22]. In the LC of normal rats, knockdown of GLT-1 alone is sufficient to increase basal extracellular glutamate concentrations [23], supporting the primary role of GLT-1 in glutamate regulation of the LC. In rats with chronic neuropathic hypersensitivity, peripheral nerve injury decreases the expression of GLT-1 via activation of histone deacetylase (HDAC) and increases basal extracellular glutamate concentrations, which reduces noxious stimulation-evoked glutamate release, via activation of presynaptic mGluRs [21]. This reduced glutamate release reduces stimulation-evoked neuronal activity in the LC and noradrenaline release in the spinal cord, thereby impairing noxious stimulation-induced analgesia [21]. This is consistent with the clinical observations of patients with established neuropathic pain having a reduced ability to physiologically recruit descending inhibition [24]. Recent laboratory studies in rats, with chronic neuropathic hypersensitivity, demonstrated that repeated administration of an HDAC inhibitor restores this impaired noxious stimulation-induced analgesia by restoring GLT-1 expression in the LC [21].

## 3. Gabapentinoids

### 3.1. LC Is an Important Target of Gabapentin Analgesia

Gabapentin, originally licensed as an antiepileptic drug in 1993, was rapidly recognized as an analgesic in patients and animals with neuropathic pain [25,26,27,28]. Gabapentin interacts with the α2δ subunit of voltage-gated calcium channels that modulates the release of excitatory amino acids in the spinal dorsal horn [29] and produces analgesia in transgenic mice with up-regulated α2δ-1 subunits, but not in normal mice [30], suggesting that the efficacy of gabapentin relies on the α2δ subunit. Thus, most laboratory studies have focused on the theory that gabapentin primarily acts on spinal pain mechanisms. However, the clinical significance of this theory could be disputed as intrathecal gabapentin at doses from 1 mg/day to 30 mg/day for three weeks failed to show clinical efficacy in patients with noncancer pain [31], despite the known efficacy of oral gabapentin in this patient population.

Tanabe et al. first reported the role of descending noradrenergic inhibition in gabapentin analgesia by demonstrating that depletion or blockade of noradrenergic signaling in the spinal cords of mice, after peripheral nerve injury, abolishes the antihypersensitivity effect of systemically administered gabapentin [25]. Similar behavioral results are also reported in various neuropathic pain rodent models, after systemic, intra-cerebroventricular, or intra-LC administration of gabapentin [26,27,28]. Gabapentin likely acts similarly in humans, because its oral administration at a dose that produces postoperative analgesia increases the noradrenaline concentration in the cerebrospinal fluid of patients with joint pain scheduled for orthopedic surgery [19]. Together, these laboratory and clinical observations suggest that descending noradrenergic inhibition plays a key role in the analgesic efficacy of gabapentin.

### 3.2. Mechanisms of LC Activation by Gabapentin

Despite its name and structural similarity to GABA, gabapentin has no direct effects on GABA receptors [32] or spinal GABA release [33]. Gabapentin, however, does affect GABA release in the brain, although its actions are controversial and likely depend on the brain site. Some studies demonstrated that gabapentin increased GABA release in rat and human brains [34,35,36], whereas other studies demonstrated a direct reduction in GABA release upon the exposure of rat cortical synaptosomes to gabapentin [37]. In the LC, gabapentin and other α2δ ligands reduce presynaptic GABA in rodents [33,38], indicating that gabapentin reduces the influence of GABA on noradrenergic neurons by which it activates descending noradrenergic inhibition.

In rodents with relatively early-stage neuropathic pain following peripheral nerve injury (within 2–3 weeks after injury), gabapentin-induced analgesia and activation of LC neurons are abolished by blocking AMPA glutamate receptors [26]. In in vivo microdialysis studies, systemic administration or local perfusion of gabapentin in rats increases extracellular glutamate concentrations in the LC, but not in the spinal cord [39]. These observations suggest that, other than reducing the influence of GABA in the LC, gabapentin also induces glutamate release in the LC to activate descending inhibition. Although GABA inhibits glutamate release via presynaptic GABA-B receptors in many sites of the brain [40,41], blocking GABA-B receptors in the LC fails to affect basal glutamate levels and the gabapentin-induced glutamate increase in rats [39], suggesting that the tonic influence of GABA on glutamatergic terminals and the effect of gabapentin on glutamate levels is either minor or absent in the LC. In cultured rat astrocytes, gabapentin and its related α2δ ligand pregabalin increase the co-transport of Na^+^ ions and glutamate via glutamate transporters and enhance the glutamate-induced intracellular Ca^2+^ response via the reverse mode of Na^+^-Ca^2+^ exchange, thus facilitating glutamate release [42]. In rats, 2–3 weeks after peripheral nerve injury, selective blockade or knockdown of GLT-1 in the LC abolishes the effects of gabapentin on glutamate levels and hypersensitivity [39,43], suggesting that GLT-1-mediated glutamate release from astrocytes is essential to the analgesic effects of gabapentin.

**Figure 2:**
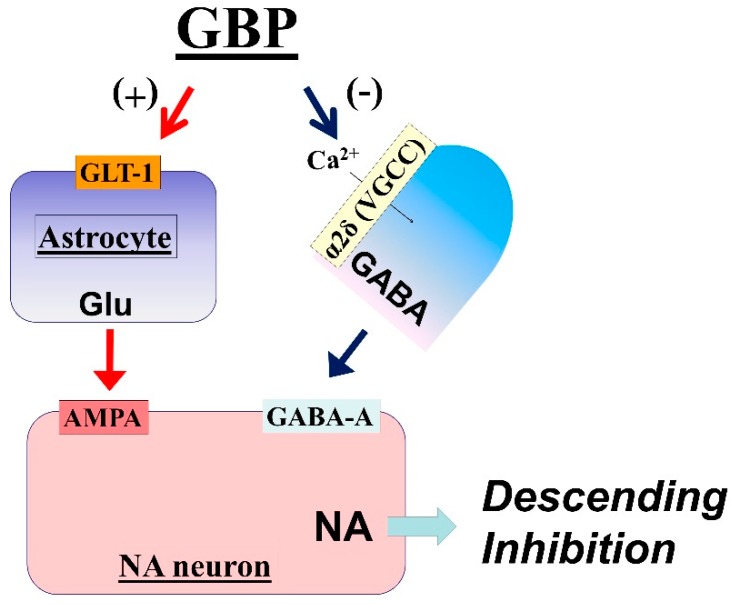
Proposed mechanisms of gabapentin (GBP) action in the locus coeruleus (LC). GBP interacts with the α2δ subunit of voltage-gated calcium channels (VGCC) to reduce presynaptic GABA release and activates glutamate transferase-1 (GLT-1)-dependent mechanisms to induce glutamate (Glu) release from astrocytes in the LC, thus increasing LC neuronal activity to activate descending inhibition.

Taken together, these observations suggest that gabapentin inhibits presynaptic GABA release and induces glutamate release from astrocytes in the LC, thereby increasing LC neuronal activity to activate descending noradrenergic inhibition, at least during the early phase (2–3 weeks after nerve injury) of neuropathic pain (Figure 2).

### 3.3. Impaired Gabapentin Analgesia in Chronic Neuropathic Pain

Gabapentin often fails to provide sufficient analgesia in patients with neuropathic pain [1], in contrast to its remarkable and uniform efficacy in various neuropathic pain rodent models [25,26,28,44]. Although there are many different factors between humans and animals regarding neuropathic pain, the discrepancy between the clinical and preclinical efficacy of gabapentin may relate in part to the timing of studies in rodents, which are typically examined within 2–3 weeks after nerve injury. Given that the analgesic effects of gabapentin rely on the expression of GLT-1 in the LC, which is down-regulated during the chronification of neuropathic pain [39,43], the early uniform antihypersensitivity effect of gabapentin decreases over time in rats after peripheral nerve injury and nearly 80% of its efficacy is lost eight weeks after injury, associated with down-regulation of GLT-1 in the LC [43]. These findings bring into question the relevance of the timing of previous laboratory studies performed within the first 2–3 weeks of surgical injury to the clinical use of gabapentin for patients with long-lasting neuropathic pain. There may be a treatment strategy to restore the impaired gabapentin-induced analgesia in chronic neuropathic pain. In rats with chronic neuropathic hypersensitivity, inhibiting HDAC by the clinically available drug valproate increases down-regulated GLT-1 expression in the LC, thereby restoring the antihypersensitivity effect of gabapentin [43]. Given the clinical availability and established safety profiles of valproate, it should be tested for rescuing gabapentin efficacy in the neuropathic pain patients who initially fail to respond to gabapentin.

## 4. Antidepressants

### 4.1. Analgesic Mechanisms of Antidepressants for Neuropathic Pain

Chronic pain causes anxiety accompanied by a depressive state and enhanced pain sensations. The analgesic effects of antidepressants on chronic pain, however, involve a mechanism different from the one which produces their antidepressive effects, because antidepressants inhibit neuropathic pain in patients without depression [45]. In addition, the effects of antidepressants on mediating antidepressive effects are visible in approximately 2–4 weeks from the time the drug is first administered, whereas the analgesic effect on chronic pain is evident in as early as a few days to one week [46].

The main pharmacologic mechanism of antidepressants involves binding with noradrenaline and serotonin (5-HT) transporters. Reuptake inhibition of these neurotransmitters leads to increased levels of noradrenaline and 5-HT in the synaptic cleft of the central nervous system [18,47,48,49]. To compare the efficacy of analgesic drugs for treating chronic pain, the “number needed to treat” (NNT) is used. The NNT represents the number of patients in whom the treatment reduced pain by as much as 50%. The lower the NNT, the stronger the efficacy. The NNT is usually obtained from meta-analysis data [50,51]. For patients with painful polyneuropathy, the NNT for noradrenaline reuptake inhibitors (e.g., nortriptyline, desipramine) is approximately 2.5 [18,52] and that for SNRIs and selective 5-HT reuptake inhibitors (SSRIs) is 5.0 and 6.8, respectively [2]. Based on these results, reuptake of noradrenaline plays a greater role than that of 5-HT in the analgesic action of antidepressants for neuropathic pain.

Several previous animal studies have demonstrated that increased noradrenaline levels in the spinal dorsal horn have an important role in the inhibition of neuropathic pain due to antidepressants [53,54,55]. Intraperitoneal administration of duloxetine, an SNRI, to rats with nerve injury inhibits hypersensitivity for at least 4 h, but the effect disappears after 24 h. The hypersensitivity gradually decreases with repeated administration of duloxetine and returns to normal levels after treatment for three consecutive days. Three daily injections of duloxetine lead to increase in noradrenaline levels in the dorsal horn of the spinal cord, and the inhibitory effect of duloxetine on hypersensitivity is reversed by intrathecal injection of an α_2_-adrenergic receptor antagonist [53]. Pretreatment with a noradrenergic neurotoxin (DSP-4) before injecting duloxetine attenuates the effect of duloxetine on antihypersensitivity [54]. Intraperitoneal administration of amitriptyline, a TCA, over consecutive days gradually suppresses hypersensitivity after nerve injury, but this antihypersensitivity effect is reversed by intrathecal injection of an α_2_-adrenergic receptor antagonist [54]. Another study demonstrated that a single intraperitoneal administration of the SNRI milnacipran in nerve-injured rats produces antihypersensitivity effects that are reversed by intrathecal administration of an α_2_-adrenergic receptor antagonist. Intrathecal injection of several types of selective 5-HT receptor antagonists, however, does not reverse the effect of milnacipran [55]. A single injection of some antidepressants, such as amitriptyline, duloxetine, milnacipran, and the SSRI fluoxetine, increases the noradrenaline level in the dorsal horn of the spinal cord [56]. In addition, noradrenaline is increased in the spinal cord by a single intraperitoneal administration of the SSRI paroxetine, and this drug produces an antihypersensitivity effect after nerve injury. The antihypersensitivity effect of paroxetine is inhibited by intrathecal injection of an α_2_-adrenergic receptor antagonist [55]. The effects of fluoxetine and paroxetine to increase noradrenaline are likely indirect because both drugs weakly inhibit noradrenaline transporters [57,58]. Intraperitoneal administration of amitriptyline, duloxetine, milnacipran, and fluoxetine at a dose of 10 mg/kg increases dopamine levels in the spinal cord and inhibits hyperalgesia in a rat model of neuropathic pain through D2-like receptors [59]. Although it is unclear why antidepressants increase dopamine levels in the spinal cord, reuptake of dopamine is mediated through noradrenaline transporters in the frontal cortex, where there are few dopamine transporters [60]. Taken together, the main mechanism of antidepressants that inhibit neuropathic pain is to increase noradrenaline in the spinal cord. Dopamine and 5-HT are also increased by antidepressants in the spinal cord and may enhance the inhibitory effects of noradrenaline in an auxiliary manner.

### 4.2. Actions of Antidepressants on the LC

The LC is characterized by both tonic and phasic neuronal activity. Phasic activity is excitatory and is observed shortly after the release of an excitatory amino acid (mainly glutamate) in the LC. Phasic activity occurring simultaneously with low to medium tonic activity is involved in attention, movement, and concentration on outside stimuli, such as cognitive functions and endogenous analgesia [21,61]. Descending noradrenergic neurons underlie endogenous analgesia. Noxious stimuli activate LC phasic activity bilaterally, inducing the release of noradrenaline through projections to the dorsal horn of the spinal cord bilaterally in rats [21,62,63].

An animal model of noxious stimulation-induced analgesia (NSIA) can be used to measure the intensity of endogenous analgesia. Mechanical stimuli applied to the hind paw after injection of capsaicin to the forepaw greatly increases the paw withdrawal threshold by activating endogenous analgesia [64]. Noradrenaline levels in the spinal cord are increased by injection of capsaicin into the forepaw, which affects NSIA [21,64,65]. This means that capsaicin-induced pain phasically activates the LC, leading to a release of noradrenaline in the spinal cord, which mediates the antinociceptive effects through α_2_-adrenergic receptors. In animal models of neuropathic pain, six weeks after nerve injury, NSIA is no longer observed, and noradrenaline levels are not increased in the spinal cord [21,53,65]. When the tonic activity of the LC is increased due to nerve injury, phasic reactivity to noxious stimuli disappears [21,53]. Phasic activity of neuronal cells in the LC gradually declines in the neuropathic pain model over a long period of time following nerve injury, and the descending noradrenergic inhibitory system is impaired.

Impaired NSIA after nerve injury in animals is recovered by the administration of duloxetine and amitriptyline over several consecutive days [53,65]. The induction of antidepressants increase the noradrenaline levels in the spinal cord, as well as their effects on the LC, contribute to NSIA recovery [53]. Several brain regions send efferents to the LC, and both noradrenaline and 5-HT mediate LC activity [7]. Antidepressants increase noradrenaline near the LC [66] and inhibit its activity through α_2_-adrenergic receptors [67,68]. One study, however, demonstrated that consecutive administration of duloxetine and desipramine increase noradrenaline, which desensitizes the α_2_-adrenergic receptors in the LC [69]. The reaction of the LC to noxious stimuli differs between animal models of neuropathic pain and control animals due to sensitization via N-methyl-D-aspartic acid receptors, but the reaction is normalized by the consecutive administration of duloxetine and desipramine [69].

BDNF and its receptor TrkB may also have important roles in strengthening impaired LC function. Chronic, but not acute, administration of antidepressants increases BDNF mRNA expression in the rat hippocampus [70]. Antidepressants increase BDNF levels in astrocyte cultures [71]. A recent study showed that repeated systemic injections of a TrkB agonist recovers weakened NSIA in rats, six weeks after nerve injury, by improving LC reactivity to noxious stimuli, and the effect was reversed by systemic injection of a TrkB antagonist [72]. Moreover, the basal extracellular glutamate concentration in the LC increases after nerve injury [21], and noxious stimulation-evoked glutamate release is decreased, thereby reducing AMPA receptor-mediated LC activation, which is important for inducing NSIA. BDNF triggers the phosphorylation of AMPA receptors and regulates AMPA receptor trafficking to the cell membrane [73]. Therefore, the impaired LC function after nerve injury may be ameliorated by antidepressants via increased BDNF levels.

## 5. Strategies to Manage Neuropathic Pain on the Basis of Animal Studies

Although clinical reviews show that gabapentinoids and antidepressants (TCA and SNRI) are first-line drugs for treating neuropathic pain [1,2,3,4], strategies to select the first drug or to combine drugs are not well established. Animal studies showed that gabapentinoids stimulate the LC resulting in activation of the noradrenergic descending inhibitory system, which impairs reactivity to noxious stimuli, after nerve injury, in a time-dependent manner. Antidepressants increase noradrenaline in the spinal cord and also act at the LC to restore its reactivity after nerve injury. Therefore, gabapentinoids should be used for patients who can recruit descending inhibition (mostly early-stage neuropathic pain). If the effect of gabapentinoids is poor or inadequate, the medication should be changed to antidepressants or a combination of gabapentinoids and antidepressants. For late-stage neuropathic pain, the HDAC inhibitor valproate might be added.

## 6. Conclusions

Both gabapentinoids and antidepressants use the noradrenergic descending inhibitory system to inhibit chronic pain, including neuropathic pain. Although several lines of evidence indicate that the function of the descending noradrenergic inhibitory system is impaired in the chronic neuropathic pain state, other recent studies report that antidepressants and HDAC inhibitors restore the impaired noradrenergic descending inhibitory system. These findings suggest new strategies to treat chronic pain by reinforcing the descending noradrenergic inhibitory system.
